# Mangroves and coastal topography create economic “safe havens” from tropical storms

**DOI:** 10.1038/s41598-021-94207-3

**Published:** 2021-07-28

**Authors:** Jacob P. Hochard, Edward B. Barbier, Stuart E. Hamilton

**Affiliations:** 1 Haub School of Environment and Natural Resources, University of Wyoming, Bim Kendall House, 804 E Fremont St, Laramie, WY 82072 USA; 2 Department of Economics, Colorado State University, Fort Collins, USA; 3 Department of Geography and Geosciences, Salisbury University, Salisbury, USA

**Keywords:** Environmental economics, Climate-change adaptation

## Abstract

Evidence suggests that mangroves protect economic activity in coastal areas. We estimate this protection from mangroves and coastal elevation globally, examining both “direct” and “indirect” exposure events (< 100 km vs. ≥ 100 km distance from a cyclone’s “eye”, respectively). We find that higher elevation (≥ 50 m) *or* wide mangroves (≥ 10 m seaward width) alone shelter economic activity from indirect cyclone exposure, whereas protection from direct cyclone exposure occurs only in high elevation communities *with* wide mangroves. Our results reveal that the majority of these “safe havens” are in upper middle-income countries but provide significant benefits to populations in lower middle-income countries.

## Introduction

Recent evidence suggests that the frequency of intense tropical cyclones has been increasing, and that this trend is likely to continue with climate change^1,2^. As a consequence, there is growing interest in the protective role of mangroves that shelter coastlines during storm events by mediating the physical impacts of storms, such as decreasing water flow pressure, storm surge height, and wind speeds while also reducing flooding levels, durations and saline water intrusion^3–8^. However, around one quarter of the world's mangroves have been lost due to human activity from 1980 to 2005^9^, mainly through conversion to aquaculture, agriculture and urban land uses^10,11^. Although annul rates of mangrove losses have slowed^12^, the global disappearance of mangroves is having a major impact on the vulnerability of coastal populations and property in developing countries, especially with respect to damaging and life-threatening storms and floods^3,7,13,14^.

A number of studies have estimated the benefits of mangroves in terms of protecting physical property, local agriculture and industry, and lives in coastal areas^15–24^. To date, most studies of the protective benefit of mangroves focus on loss of economic activity and property, and only a few estimate any resulting impacts on either the disutility from risk aversion or the risk of possible injury, illness or death as a result of cyclones. One study estimates that, during the 1999 cyclone that struck Orissa, India, there would have been 1.72 additional deaths per village within 10 km of the coast if mangroves had been absent^17^. An analysis for the 2004 Indian Ocean tsunami finds that mangroves, forests and plantations situated between villages and the coastline in Aceh, Indonesia may have decreased loss of life by 3% to 8%^20^. A global study estimates individual-level exposure through applying a damage function model with local variation in flood depths and gridded population maps^23^.

Overall, current studies suggest that there are three important spatial aspects of the protective benefits of mangroves. First, the presence of mangroves, especially in low-lying coastal villages, may mitigate otherwise permanent loss of economic activity due to direct cyclone exposure, and this protection is affected by differences in widths of seaward mangrove found along coastlines^15,16,19,22,24,25^^.^ Second, even in low-lying coastal zones, difference in elevation may matter, in that households and communities located in higher coastal elevations may be less vulnerable to cyclone damage and may not necessarily require more protection from mangroves^17,20,23^. Finally, the role of both mangroves and coastal elevation may depend on cyclone exposure; that is, households and communities in the direct path of the storm experience more damaging storm impacts, such as rainfall, flooding extent, wind speeds, storm intensity, etc.^17,21,26^. The purpose of the following paper is to examine the influence of these spatial aspects on the protective benefits of mangroves and coastal topography in sheltering against cyclone damage to coastal property and economic activity.

We focus on a coastal population of nearly 400 million individuals that are prone to tropical cyclones from 2000 to 2012. The analysis tracks 2549 coastal mangrove-holding communities within 102 countries. We present the 5-year sustained impact of tropical storm exposures on nighttime luminosity trends to evaluate long-run disruptions in economic activity caused by tropical storms. A series of econometric specifications and tests reveal the relative roles of coastal topography and mangrove forests in mediating these economic disruptions. The combination of coastal topography and mangroves that shelter coastal economic activity from direct and indirect impacts is then discussed in the context of global, regional and country-level and subnational distributions of income and levels of economic development.

## Results

### Empirical findings

We stratify cyclone exposure intensities into three bins. Bin 1 represents “direct” exposure and includes administrative units that were located within 100 km of the cyclone’s “eye”. Bins 2 and 3 represent “indirect” exposure and include administrative units that were situated within 100 km to 200 km and 200 km to 300 km of the cyclone’s “eye”, respectively. We consider administrative units located within the latter distances to the cyclone’s eye to be subject to “indirect” exposure, because these areas represent locations that were likely exposed to lower wind intensities and shorter exposure durations. Within each bin of exposure, we further stratify our sample into four subsamples: low and narrow, high and narrow, low and wide and high and wide. “Low” selects administrative units into the subsample if the mean elevation is less than 50 m and “narrow” selects administrative units into the subsample if their average mangrove width is less than 10 m of mangroves from the seaward boundary per m of coastline. Consequently, “high” corresponds to a mean elevation of 50 m or more, and “wide” is average mangrove extent equal to or greater than 10 m per m of coastline.

**Figure 1:**
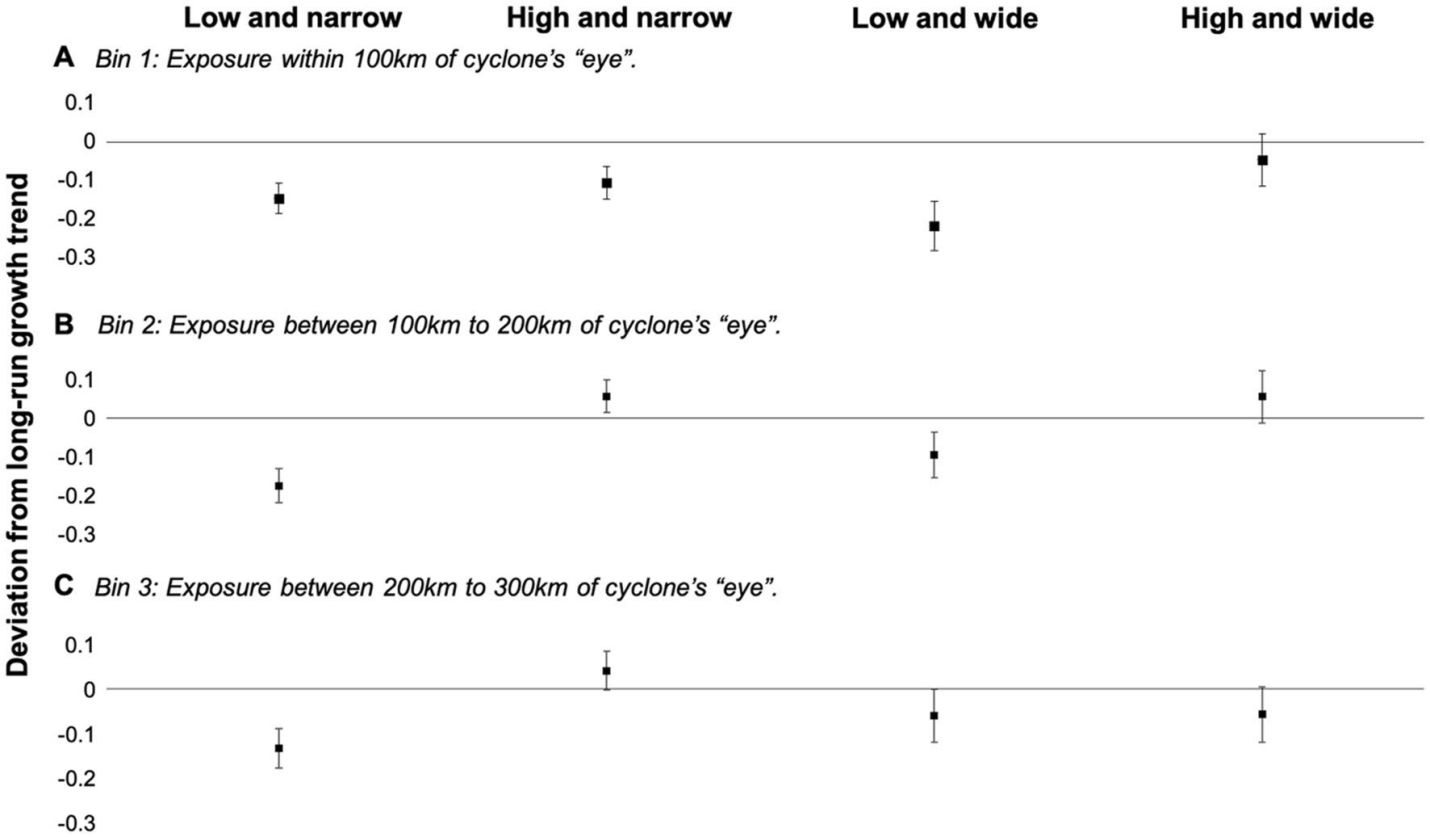
Five-year cumulative impact of tropical cyclone exposure on the growth trend in economic activity. (**A**) represents direct exposure within 100 km of a cyclone’s “eye” and (**B**,**C**) represent indirect exposure from 100 to 200 km of a cyclone’s and 200 km to 300 km of a cyclone’s “eye”. “Low” includes administrative units with a mean elevation < 50 m and “high” corresponds to mean elevation ≥ 50 m. “Narrow” includes administrative units with average mangrove width < 10 m of mangroves from the seaward boundary per m of coastline, and “wide” is average mangrove width ≥ 10 m per m coastline. 95% confidence intervals are provided around point estimates.

The cumulative impact of cyclone exposure on economic activity is measured across five annual lags for all 12 datasets (3 bins each with 4 subsamples). For example, the fifth lag (representing the exposure year and four years into the future) for bin one and subsample 1 captures the persistent and cumulative impact of direct cyclone exposure on economic activity in low-lying administrative units that have narrow mangrove coverage (Fig. 1A).

We find that direct exposure has a permanent impact on long-run economic outcomes in the absence of natural protections that buffer winds or reduce storm surge inundation (Table 1). Five years following exposure, point estimates show a 10% to 22% loss in economic activity when compared to pre-cyclone trends (Fig. 1A). However, for those coastal communities at high elevation with wide mangroves, we estimate a statistically insignificant point effect of − 4.6% (Fig. 1A). Whereas the cumulative effect of exposure on “unprotected” communities continues to worsen in the fifth year, results suggest that cyclone exposure disrupts economic activity in coastal communities in the year of impact *and* in subsequent years following exposure (Table 1). High elevation and wide mangroves, together, buffer this initial impact of storm exposure and enable communities to return to pre-exposure growth rates quickly, thus avoiding long-term impacts on economic activity. In the case of direct exposure, elevation or mangroves alone appear incapable of sheltering economic activity.For indirect exposure, we find that mangroves or elevation alone have the capacity to generate tremendous storm protection benefits (Fig. 1B,C and Table 1). For those exposed between 100 and 200 km of the cyclone’s eye, low elevation communities with narrow mangroves experienced a 17.0% reduction in long-run economic growth, which was reduced to 9.0% for similarly low elevation communities with wide mangroves (Fig. 1B). Likewise, for those communities exposed between 200 and 300 km of the cyclone’s eye, low elevation communities with narrow mangroves experienced a 12.9% reduction in long-run economic growth, which was reduced to 5.5% for similarly low elevation communities with wide mangroves (Fig. 1C). For these indirect exposures, high elevation has a substantial benefit for those communities with narrow mangroves, but this benefit from being at high elevation appears dampened for those coastal communities that already have wide mangroves.

**Table 1:** Five-year cumulative effects of cyclone exposure on growth rate in economic activity across four subsamples (low and high elevation coastal communities, narrow and wide mangroves) and three bins of direct and indirect exposure (“eye” of the storm passing within 100 km, 100–200 km and 200–300 km of coastal community).

$\beta$ vector	Subsample 1: low and narr	Subsample 2: high and narr	Subsample 3: low and wide	Subsample 4: high and wide
**Bin 1 (0 to 100 km)**				
Lag 0—impact year	− 0.0459***	− 0.0164**	− 0.0547***	− 0.0193
+ Lag 1	− 0.0835***	− 0.0354***	− 0.1165***	− 0.0297
+ Lag 2	− 0.1151***	− 0.0633***	− 0.1624***	− 0.0354
+ Lag 3	− 0.1398***	− 0.0877***	− 0.1953***	− 0.0437
+ Lag 4	− 0.1456***	− 0.1046***	− 0.2172***	− 0.0459
**Bin 2 (100 to 200 km)**				
Lag 0—impact year	− 0.0472***	0.0056	− 0.0341***	0.0065
+ Lag 1	− 0.0903***	0.0144	− 0.0618***	− 0.0112
+ Lag 2	− 0.1346***	0.0360**	− 0.0893***	− 0.0043
+ Lag 3	− 0.1515***	0.0568***	− 0.1092***	0.0278
+ Lag 4	− 0.1698***	0.0616***	− 0.0900***	0.0599
**Bin 3 (200 to 300 km)**				
Lag 0—impact year	− 0.0354***	0.0030	− 0.0294***	− 0.0247**
+ Lag 1	− 0.0667***	0.0048	− 0.0411***	− 0.0584***
+ Lag 2	− 0.1021***	0.0235	− 0.0585***	− 0.0774***
+ Lag 3	− 0.1139***	0.0398**	− 0.0691***	− 0.0727***
+ Lag 4	− 0.1290***	0.0460**	− 0.0551***	− 0.0522
Fixed effects—year	Y	Y	Y	Y
Fixed effects—community	Y	Y	Y	Y
Observations	12,005	6294	6719	2751
R-squared	0.8271	0.8399	0.8376	0.8710
Root MSE	0.1431	0.1224	0.1444	0.1157

All specifications report cumulative effects with robust standard errors, four autoregressive lags in addition to the year of exposure, two forward lags on cyclone exposure and controls for mangrove width and the baseline logged growth rate. “Low” sub-samples contain coastal communities with a mean elevation < 50 m, and “high” have a mean elevation ≥ 50 m. “Narrow” sub-samples contain coastal communities with an average mangrove width from the seaward boundary of < 10 m per m of coastline, and “wide” an average mangrove width of ≥ 10 m per m of coastline. ∗∗p < 0.05, ∗∗∗p < 0.01.

For indirect exposures, it appears storm protection services from mangroves are substitutable for increased elevation, whereas for direct exposures, it appears mangroves and elevation are necessary complements to sheltering long-run economic growth from cyclones. Importantly, for all direct exposure specifications, including the case of combined elevation and mangrove protections, the cumulative effect continues to increase, albeit taper off in magnitude, 5 years following exposure.

### Distributional analysis

With climate predictions of increased frequency of intense storm events, our work presents three key findings. First, low-lying coastal areas require wide mangrove forests of at least 10 m width from the seaward boundary per m of coastline to buffer long-term losses to economic activity—narrow bands of mangroves provide little protection. Second, even wide mangroves are only capable of sheltering those communities exposed indirectly to cyclones. Third, for coastal safe havens to be sheltered against increasingly intense storm events requires both high elevation (≥ 50 m) and wide mangroves.

Although our sample contains nearly 400 million individuals with mangroves along the coastline of their community (Tables 2 and S1A–D), approximately 60 million have wide enough mangroves in low-lying areas to generate protection from an indirect exposure event (Table S1C). These communities are located overwhelmingly in lower-middle income countries (38.4%) and within developing countries in Latin America and Caribbean (34.0%) and East Asian and Pacific (32.8%) countries. We estimate only 10.9% of this population is in the developed world. These coastal communities within lower-middle income countries appear to be concentrated behind a relatively small share of the world’s expansive mangroves (34.7%) compared to upper-middle income countries that have 10.9% of the sample population but 52.4% of the mangrove coverage.

**Table 2:** 2010 sample total population and mangrove coverage summary statistics for full sample of 102 territories and countries and 2549 coastal communities.

	Total pop.	Total pop. (%)	Mangrove coverage (m^2^)	Mangrove coverage (%)
Global	384,236,006	100.0	1,898,459,072	100.0
**Developing regions**				
East Asia and Pacific	173,720,261	45.2	543,918,977	28.7
Latin America and Caribbean	57,253,635	14.9	716,289,048	37.7
Middle East and North Africa	1,980,497	0.5	20,745	0.0
North America	0	0.0	0	0.0
South Asia	65,931,107	17.2	22,434,918	1.2
Sub-Saharan Africa	27,736,477	7.2	313,254,661	16.5
**Income categories**				
High income	56,967,111	14.8	202,499,101	10.7
Upper-middle income	112,058,912	29.2	1,006,538,398	53.0
Lower-middle income	180,439,252	47.0	565,763,614	29.8
Low income	12,136,753	3.2	23,601,736	1.2
**Developed vs. developing**				
Developed	57,711,514	15.0	203,254,528	10.7
Developing	326,524,492	85.0	1,595,903,748	84.1

Country-level data are included in supplementary materials.

Categories are based on World Bank Country and Lending Groups current for the 2019 fiscal year (see https://datahelpdesk.worldbank.org/knowledgebase/articles/906519-world-bank-country-and-lending-groups). Low income countries are those with a Gross National Income (GNI) per capita less than $995, lower-middle income countries have a GNI/pp between $996 and $3895, upper-middle income countries have a GNI/pp between $3896 and $12,056 and upper-middle income countries have a GNI/pp > $12,056. Developing regions exclude countries with high income in their aggregations.

Even in the absence of wide mangrove coverage, high elevation is sufficient to generate protection from an indirect exposure event. Such high elevation coastal communities without wide mangrove coverage account for approximately 140 million people globally (Table S1B). Over 75% of this population is located within developing countries with the majority located in East Asia and Pacific developing countries (55.4%). Despite 49.1% of “narrow” mangroves in high elevation areas being located in Latin America and Caribbean developing countries, these areas only account for 11.5% of the relevant global population. These high elevation and narrow mangrove populations are evenly distributed among lower-middle income (38.4%) and upper-middle income (35.5%) countries. However, 55.2% of such mangrove coverage is located within upper-middle income countries as compared to 25.7% in lower-middle income countries.

Protection from direct exposure events requires high elevation and wide mangrove coverage. We find that 28% of global mangrove coverage (approximately 504 million m^2^) has a seaward width more than 10 m along communities with mean elevation > 50 m (Table S1D). These areas represent “safe havens” from direct and indirect exposure from cyclones and are located overwhelmingly in upper-middle income countries (65.7%). Yet, 47.2% of populations in these areas reside in lower-middle income countries whereas only 45.4% of populations in these areas reside in upper-middle income countries (Table S1D). Based on population (93.9%) and mangrove coverage (93.2%) distributions, we find that these “safe havens” are located disproportionately in developing countries.

We find that the majority of people receiving coastal topographic and mangrove protections from indirect storm exposures are concentrated in few countries. Coastal populations with low elevation and wide mangroves are located overwhelmingly in Indonesia (18.7%), Brazil (15.4%), Nigeria (10.9%), Malaysia (10.0%) and USA (9.0%), which together account for 63.9% of the global population in such areas. Coastal populations with high elevation and narrow mangroves are concentrated in Indonesia (30.9%), China (22.0%), Saudi Arabia (10.2%), Malaysia (7.0%) and Mexico (4.5%), which together account for 74.5% of the global population in such areas (country-level data provided in supplementary materials).

Coastal “safe havens” that provide the unique combination of topography and mangrove coverage to shelter against direct storm exposures are also concentrated among few countries. Indonesia (41.7%) and Brazil (11.8%) have over 15.8 million people accounting for 53.5% of the global population in such areas. Including Malaysia (9.8%), Mexico (6.9%) and Jamaica (3.2%) drives the global share of “safe haven” populations to 73.4%, or 21.8 million individuals. Although Indonesia contains the largest “safe haven” population, the within-country distribution of this population is also concentrated. The four regencies (second-level administrative subdivision) with the largest “safe haven” populations are Cilacap, Deli Serdang, Banyuwangi and Sumenep, which, account for 6.7 million people or 54.4% of the Indonesian “safe haven” population. Together, these four Indonesian regencies alone represent 22.7% of the global “safe haven” population.

## Discussion

Our findings suggest that mangroves shelter coastal economic activity for over 60 million individuals that are prone to indirect cyclone exposures in low-lying areas. These locations are predominately in developing countries. Direct cyclone exposure, which is measured by storm impact within 100 km of the cyclone’s “eye”, leaves a permanent and detrimental impact on economic activity in such communities. Further, this vulnerability to direct storm exposure is likely to persist and potentially amplify as future storms intensify. Whereas mangrove conservation efforts today may shelter coastal communities from future and less intensive storms and indirect exposure events, out-migration to elevated areas with expansive mangroves may be needed to protect coastal communities against future exposures.

It has been long recognized that global mangrove distributions are concentrated in a few developing countries, such as Indonesia, Brazil, Nigeria and Malaysia. However, our findings suggest that coastal “safe haven” locations, characteristic of high elevation ($\ge 50 \, \text{m}$ elevation) and wider mangroves ($\ge 10\, \text{m}$ mangroves/m of coastline), may be even more concentrated than the existing global distribution of mangroves. Globally, Indonesia has the largest coastal populations with wide mangroves in low-lying areas *and* with wide mangroves in high elevation areas. However, Indonesia’s global population share in high elevation, or “safe haven”, areas is twice as large as its global population share in low-lying areas. Within Indonesia, the majority of the “safe haven” population are located in only four of the country’s 36 relevant regencies.

These “safe haven” areas are overwhelmingly in upper-middle income countries but are used disproportionately by lower middle-income populations. This trend may represent a stronger dependence of poorer, therefore more vulnerable, populations on natural infrastructure for storm protection. In such a case, we might expect migration to such areas following a large storm exposure event in lower middle-income countries. However, for upper middle-income countries, communities may substitute built infrastructure for natural infrastructure in low-lying areas, using shoreline armoring initiatives (e.g., sea walls, groins, jetties, etc.) that provide alternative storm protection services than elevation and expansive mangroves.

The substitutability, in terms of protecting coastal communities from storm events, between shoreline armoring and mangrove conservation is not the focus of this work but is essential to drawing policy conclusions. Similarly, our analysis is unable to capture a longer-term economic rebound effect that might occur beyond our 5-year analysis window. While it appears these disruptive effects to economic activity are “tapering off”, the relevance of 10-year and 20-year dynamic effects following cyclone exposure remain unclear.

Future work should focus on whether the sparsely populated safe havens in middle income-countries serve as an “option” for future adaptation or a redundancy with other built infrastructure investments. Globally georeferenced datasets documenting built coastal adaptation infrastructure alongside improved coastal elevation modeling (for example, MERIT-DEM) would make such a nuanced analysis viable. Such a higher resolution analysis may also lead to a better understand of the joint production of storm protection services along a finer continuum of coastal topographic and mangrove coverage changes. Such work should consider whether further damages to economic activity increase after 5 years or whether a delayed economic recovery phase ensues, with above normal growth in economic activity that may offset these otherwise permanent losses.

## Methods

### Data construction

We construct an annual panel dataset from 2000 to 2012 of 2549 coastal communities within 102 countries. Population counts from 2000 to 2012 for each community were calculated from the Landscan population database^27^ and coastal communities were defined as the lowest level administration units with an ocean coastline of each country using the Global Administrative Areas Database v2.7. Using the National Oceanic and Atmospheric Administration’s (NOAA) global nighttime lights data, we examine trends in economic activity before and after a cyclone event. The growth rate in average annual luminosity from nighttime lights trends with economic growth and has been used as an effective proxy for local economic activity^22,24,28–32^.

However, trends in nighttime luminosity should not be interpreted as a measure of economic growth. Instead, we focus on tracking the dynamic impacts of nighttime luminosity (e.g. deviations from trends) that indicates whether an exposed community’s economic activity recovers or suffers permanent damage. The average elevation of each coastal community was calculated using a void-filled Shuttle Radar Topography Mission (SRTM) data at 3 arc-seconds, or approximately 90 m^2^ at the equator^33^. The SRTM has the potential to result in an overestimation of elevation in heavily built environment areas or areas of dense high forest canopies compared against locations without such trees. However, during the timeframe of our analysis, the SRTM product was the most appropriate and available product.

The mangrove coverage dataset was adapted from the Continuous Global Mangrove Forest Cover for the 21st Century (CGMFC-21) database for the years 2000 to 2012^12^. The coastline length of each community, based on Global Self-Consistent, Hierarchical, High-Resolution Shoreline Database v2.3.5 full resolution data^34^, was used to normalize the area of mangroves offshore of each coastal community creating a measurement for the “width” of mangroves per meter of coastline.

Tropical storm locations for all years were recreated from the International Best Track Archive for Climate Stewardship (IBTrACS) Annual Tropical Cyclone Best Track Database^35^*.* Precise measurements of exposure, combined with high-resolution luminosity data, allows to distinguish the heterogeneous impacts of cyclones on exposed communities and the capacity for mangroves to shelter coastal economic activity at different elevations and for different mangrove widths. The intensity of exposure is measured by the distance of the cyclone’s “eye” from the exposed village’s nearest boundary.

Tropical cyclone wind profile^36^, villages passing within 100 km of the cyclone’s eye were likely to experience maximum wind velocity *and* surface level pressure whereas those villages passing within more distant bands—i.e., 100–200 km and 200–300 km, were likely to experience similar surface level pressure but a non-linear reduction in wind velocity. Binning wind velocities in this way recognizes the highly non-linear relationship between wind velocity and on-the-ground damages from cyclone events^37^. We therefore expect the capacity for mangroves and elevation to shelter economic activity also to depend on this intensity of exposure.

Our full sample encompasses nearly 400 million individuals in 102 countries and 2549 mangrove-holding communities (Table 1). Based on 2019 fiscal year World Bank categorizations, most of our sample resides in developing countries (85.1%) with 46.7% in lower-middle income (gross national income/per capita between $996 and $3895) and 35.3% in upper-middle income countries (gross national income/ per capita between $3896 and $12,056). We also find that most mangrove coverage in our sample exists within developing countries (88.7%) and overwhelmingly in upper-middle income countries (56.0%) in the Latin America and Caribbean (LAC) and East Asian and Pacific (EAP) developing regions. While only 14.9% of our sample’s global population resides in LAC countries, these countries account for 39.8% of mangrove holdings in our sample whereas the 45.5% of the population residing in EAP countries only account for 30.3% of mangrove coverage.

### Empirical strategy

We use a distributed-lag autoregressive model to measure the initial and permanent effect of cyclone exposure on economic activity in coastal communities. The growth in economic activity for each coastal community is proxied by the difference in logs between years, $growth={\ln}\left(luminosit{y}_{t}\right)-{\ln}\left(luminosit{y}_{t-1}\right)$. Our estimating equation is

1 $$growt{h}_{i,j,t}=\sum\limits_{L=0}^{n}{[\beta }_{L} x {C}_{i,j,t-L}]+{\gamma }_{j}+{\delta }_{t}+\eta {X}_{i,j,t}+{\epsilon }_{i,j,t}$$

where the $\beta$ coefficients capture the marginal effects, across three bins of cyclone exposure, on the growth rate of luminosity for the $j{^{\prime}}th$ administrative unit, within country $i$, and in time $t-L$ where $t$ is the observed year and L is the number of lags ranging from $0 \; to \;n$. Here, ${C}_{i,j,t}$ is a vector of cyclone exposures binned by the distance from the cyclone’s “eye” to the nearest boundary of the exposed community (< 100 km, 100–200 km and 200–300 km). The distributed-lag autoregressive model predicts $growt{h}_{i,j,t}$ using a vector of cyclone exposures in previous, current and future years—i.e., forward and backward lags. Such an approach isolates the marginal impacts of a cyclone exposure on growth trends without being confounded by multiple reoccurring exposures.

We adopt community-specific and year-specific fixed effects to control for any unobservable impacts, captured by $\gamma$ and $\delta$, on economic activity for a given community or within a given year. Mangrove width and the logged baseline level of luminosity (digital number units—i.e., DN) are added as control variables in the vector, ${x}_{i,j,t},$ as well as a linear trend to absorb background growth trends that are shared by communities in our sample. Four autoregressive lags are also included in all specifications and robust standard errors are reported.

**Figure 2:**
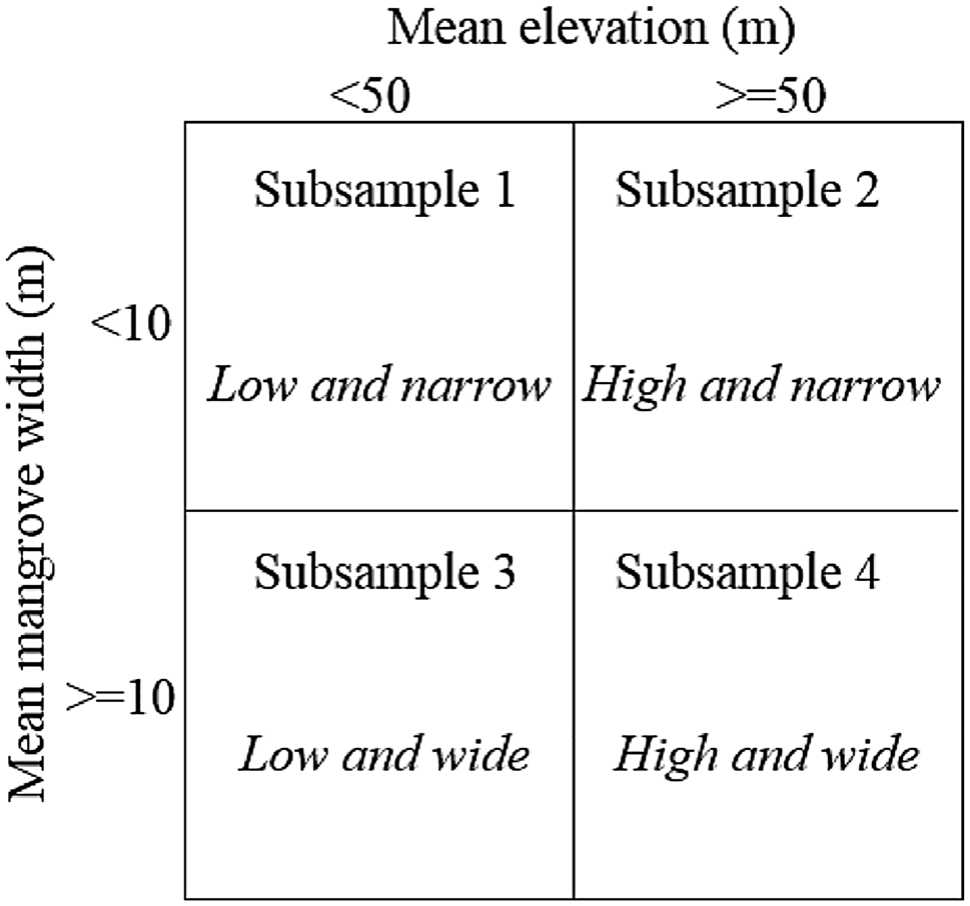
Subsamples for elevation and mangrove width stratifications.

The impact of a cyclone on long-run trends in economic activity $z$ years later is

$${\Lambda }_{i,j}={\sum }_{L=0}^{z}{[\beta }_{L}],$$

which is cumulative effect (summation of marginal effects) of cyclone exposure on luminosity growth. To examine the scope for mangroves of varying width and topography of varying elevation to shelter coastal economic activity, we stratify our sample into four subsamples: “low and narrow”, “low and wide”, “high and narrow” and “high and wide”. “Low” sub-samples contain coastal communities with a mean elevation < 50 m and “narrow” sub-samples contain coastal communities with a mangrove width of < 10 m per meter of coastline (Fig. 2).

The 50 m elevation and 10 m mangrove distance thresholds were chosen to provide some balance in statistical power to our four subsamples. Here, the unit of observation in our analysis is the lowest available administrative unit. A location with an average coastal elevation of 50 m elevation is likely to have a large and vulnerable population living in low-lying areas. Similarly, a coastline with an average of 10 m of mangroves per m of coastline is likely to have vast expanses of mangroves in some of its locations. However, the average area of our unit of observation is 887 square kilometers and the average administrative unit’s coastline length is 32 km. As such, those low-lying populations and expansive mangroves get absorbed into the aggregation process rather quickly. These thresholds are only meant to facilitate the binning of our sample into four groups (binary low vs. high elevation, binary narrow vs. wide mangroves).

We hypothesize that communities in subsample 1, lacking natural protections against storm exposure, are the most vulnerable and would experience the strongest effect on long-run economic outcomes. We further hypothesize that communities in subsample 4, receiving protection from mangroves and topography, would be the most insulated against storm exposures. Likewise, we would expect communities benefiting from either expansive mangroves or high elevation, would be partially protected from exposure and adverse long-run economic impacts. In terms of “intensity of exposure”, we hypothesize that those communities passing within the nearest proximity to the cyclone’s “eye” will experience the largest adverse impact on economic growth—i.e., bin 1 > bin 2 > bin 3.

## Supplementary Information

Supplementary Information 1.

Supplementary Information 2.
