# Supplementary material for: Mangroves and coastal topography create economic “safe havens” from tropical storms
Source: Sci Rep. 2021 Jul 28;11:15359. doi: 10.1038/s41598-021-94207-3 (PMC8319195; doi:10.1038/s41598-021-94207-3)
Supplement: Supplementary file 1 — Supplementary Information 1. [file 41598_2021_94207_MOESM1_ESM.pdf]

Supplementary Information

Table S1 A: 2010 sample summary statistics for low & narrow subsample 1 including 76 territories and countries and 1,112 coastal communities.

	Total pop.	Total pop. (%)	Mangrove coverage (m^2)	Mangrove coverage (%)
**Global**	154,121,624	100.0%	54,820,827	100.0%
Developing Regions				
East Asia and Pacific	60,340,726	39.15%	10,984,221	20.04%
Latin America and Caribbean	10,285,411	6.67%	21,556,890	39.32%
Middle East and North Africa	364,711	0.24%	15,871	0.03%
North America	0	0.00%	0	0.00%
South Asia	54,568,303	35.41%	3,702,583	6.75%
Sub-Saharan Africa	13,615,483	8.83%	2,734,140	4.99%
Income Categories				
High Income	14,891,174	9.66%	15,568,248	28.40%
Upper-Middle Income	42,269,050	27.43%	24,315,119	44.35%
Lower-Middle Income	89,528,176	58.09%	13,076,158	23.85%
Low Income	7,279,923	4.72%	1,587,827	2.90%
Developed vs. Developing				
Developed	15,044,475	9.76%	15,841,723	28.90%
Developing	139,077,149	90.24%	38,979,104	71.10%

Note: Categories are based on World Bank Country and Lending Groups current for the 2019 fiscal year. Low-income countries are those with a Gross National Income (GNI) per capita less than $995, lower-middle income countries have a GNI/pp between $996 and $3,895, upper-middle income countries have a GNI/pp between $3,896 and $12,056 and upper-middle income countries have a GNI/pp >$12,056. Developing regions exclude countries with high income in their aggregations.

Table S1 B: 2010 sample summary statistics for high & narrow subsample 2 including 61 territories and countries and 577 coastal communities.

	Total pop.	Total pop. (%)	Mangrove coverage (m^2)	Mangrove coverage (%)
**Global**	140,118,617	100.0%	44,498,810	100.0%
Developing Regions				
East Asia and Pacific	77,587,438	55.37%	13,092,035	29.42%
Latin America and Caribbean	16,058,742	11.46%	21,826,236	49.05%
Middle East and North Africa	1,615,786	1.15%	4,874	0.01%
North America	0	0.00%	0	0.00%
South Asia	7,885,380	5.63%	507,193	1.14%
Sub-Saharan Africa	2,686,838	1.92%	833,442	1.87%
Income Categories				
High Income	33,885,987	24.18%	7,753,078	17.42%
Upper-Middle Income	49,738,696	35.50%	24,568,522	55.21%
Lower-Middle Income	53,765,113	38.37%	11,421,308	25.67%
Low Income	2,330,375	1.66%	273,950	0.62%
Developed vs. Developing				
Developed	34,284,433	24.47%	8,235,030	18.51%
Developing	105,834,184	75.53%	36,263,780	81.49%

Note: Categories are based on World Bank Country and Lending Groups current for the 2019 fiscal year. Low-income countries are those with a Gross National Income (GNI) per capita less than $995, lower-middle income countries have a GNI/pp between $996 and $3,895, upper-middle income countries have a GNI/pp between $3,896 and $12,056 and upper-middle income countries have a GNI/pp >$12,056. Developing regions exclude countries with high income in their aggregations.

Table S1 C: 2010 sample summary statistics for low & wide subsample 3 including 44 territories and countries and 605 coastal communities.

	Total pop.	Total pop. (%)	Mangrove coverage (m^2)	Mangrove coverage (%)
**Global**	60,319,584	100.0%	1,801,499,731	100.0%
Developing Regions				
East Asia and Pacific	19,774,557	32.78%	315,065,268	26.35%
Latin America and Caribbean	20,508,593	34.00%	478,746,329	40.04%
Middle East and North Africa	0	0.00%	0	0.00%
North America	0	0.00%	0	0.00%
South Asia	3,226,796	5.35%	7,029,840	0.59%
Sub-Saharan Africa	10,240,435	16.98%	249,932,650	20.90%
Income Categories				
High Income	6,376,547	10.57%	144,877,151	12.12%
Upper-Middle Income	6,569,203	10.89%	626679056.1	52.41%
Lower-Middle Income	23,131,744	38.35%	414,258,137	34.65%
Low Income	2,159,859	3.58%	9,836,894	0.82%
Developed vs. Developing				
Developed	6,569,203	10.89%	144,877,151	12.12%
Developing	53,750,381	89.11%	1,050,774,087	87.88%

Note: Categories are based on World Bank Country and Lending Groups current for the 2019 fiscal year. Low-income countries are those with a Gross National Income (GNI) per capita less than $995, lower-middle income countries have a GNI/pp between $996 and $3,895, upper-middle income countries have a GNI/pp between $3,896 and $12,056 and upper-middle income countries have a GNI/pp >$12,056. Developing regions exclude countries with high income in their aggregations.

Table S1 D: 2010 sample summary statistics for high & wide subsample 4 including 39 territories and countries and 255 coastal communities.

	Total pop.	Total pop. (%)	Mangrove coverage (m^2)	Mangrove coverage (%)
**Global**	29,676,181	100.0%	504,187,400	100.0%
Developing Regions				
East Asia and Pacific	16,017,540	53.97%	204,777,453	40.62%
Latin America and Caribbean	10,400,889	35.05%	194,159,593	38.51%
Middle East and North Africa	0	0.00%	0	0.00%
North America	0	0.00%	0	0.00%
South Asia	250,628	0.84%	11,195,302	2.22%
Sub-Saharan Africa	1,193,721	4.02%	59,754,429	11.85%
Income Categories				
High Income	1,813,403	6.11%	34,300,624	6.80%
Upper-Middle Income	13,481,963	45.43%	330,975,701	65.65%
Lower-Middle Income	14,014,219	47.22%	127,008,011	25.19%
Low Income	366,596	1.24%	11,903,065	2.36%
Developed vs. Developing				
Developed	1,813,403	6.11%	34,300,624	6.80%
Developing	27,862,778	93.89%	469,886,776	93.20%

Note: Categories are based on World Bank Country and Lending Groups current for the 2019 fiscal year. Low-income countries are those with a Gross National Income (GNI) per capita less than $995, lower-middle income countries have a GNI/pp between $996 and $3,895, upper-middle income countries have a GNI/pp between $3,896 and $12,056 and upper-middle income countries have a GNI/pp >$12,056. Developing regions exclude countries with high income in their aggregations.
